# Effects of Ankle Joint Motion on Pelvis-Hip Biomechanics and Muscle Activity Patterns of Healthy Individuals in Knee Immobilization Gait

**DOI:** 10.1155/2019/3812407

**Published:** 2019-10-15

**Authors:** Xinyu Guan, Shengzheng Kuai, Liang Song, Weifeng Liu, Yali Liu, Linhong Ji, Rencheng Wang

**Affiliations:** ^1^Division of Intelligent and Bio-Mimetic Machinery, The State Key Laboratory of Tribology, Tsinghua University, Beijing, China; ^2^Shenzhen Second People's Hospital, Shenzhen, China; ^3^First Affiliated Hospital of Shenzhen University, Shenzhen, China; ^4^Shenzhen University School of Medicine, Shenzhen, China; ^5^Key Laboratory of Technical Aids Analysis, Identification Key Laboratory of the Ministry of Civil Affairs, Key Laboratory of Rehabilitation Technical Aids for Old-Age Disability, National Research Center for Rehabilitation Technical Aids, Beijing, China; ^6^Department of Mechanical and Electrical Engineering, Beijing Institute of Technology, Beijing, China

## Abstract

The purpose of the study was to investigate the pelvis-hip biomechanics and trunk and lower limb muscle activity patterns between healthy people walking in two gaits and evaluate the effects of ankle joint motion on these two gaits. The two gaits included walking with combined knee and ankle immobilization and with individual knee immobilization. Ten healthy participants were recruited and asked to walk along a 10 m walk away at their comfortable speeds in the two gaits. Kinematic data, ground reaction force, and electromyography waveforms of trunk and lower limb muscles on the right side were collected synchronously. Compared to individual knee immobilization gait, people walking in the combined knee and ankle immobilization gait increased the range and average angle of the anterior pelvic tilt during the first double support and the single support phase, respectively. The combined knee and ankle immobilization gait also increased the range of hip abduction during the second double support phase. These kinematic alternations caused changes in trunk and lower limb muscle activity patterns. The ankle immobilization increased the range of gluteus maximus activation in the first double support phase, the range of rectus abdominis activation, the average amplitude of rectus femoris activation in the single support phase, and the range of rectus femoris activation in swing phase and decreased the range of and tibialis anterior activation in the first double support phase. The ankle immobilization also increased the average values of proximodistal component in AKI gait during the single support phase. This study revealed significant differences in pelvis-hip biomechanics and trunk and lower limb muscle activity patterns between the two gaits.

## 1. Introduction

Motor neuron injuries, quadriceps weakness, or postsurgical procedures may affect the walking ability of individuals. Consequently, these individuals are usually prescribed a lower limb orthosis or a brace to assist locomotion. The knee joints of the walking assistance devices (e.g., knee-ankle-foot orthosis (KAFO) and reciprocating gait orthosis (RGO)) are locked to prevent the knee from collapsing in ambulation [1]. There are a variety of ankle joints available, including solid or hinged joint. The solid joint constrains ankle plantar/dorsiflexion at 0°, and the hinged one provides ankle joint with a range of motion.

The biological ankle complex comprises the talocrural joint, the subtalar joint, and the distal tibiofibular syndesmosis [2]. The talocrural joint can be regarded as a hinged joint, allowing for plantar flexion and dorsiflexion. In the sagittal plane, articulation produces a large burst of work and is most performed at terminal stance to propel the body forward [3, 4]. The subtalar joint is a plane joint, contributing to inversion and eversion. In the frontal plane, articulation allows the foot to accommodate the environments [5], and it is related to postural control and maintaining balance [6]. Walking is a complex process involving the coordination of joint motion; thus, the motion of ankle joint can affect other joints' function and performance when walking with knee immobilization.

A few previous studies studied the effects of ankle joint motion on the gait in situation of walking with knee immobilization. For example, in comparison to RGO with solid ankle joint, Arazpour et al. found that RGO incorporating dorsiflexion-assisted ankle joint can increase walking speed and distance and reduce the physiological cost index of people with spinal cord injury [7]. Bani et al. supplemented that the hip joint range of motion (ROM) increased moderately [8]. Also, Genda et al. reported that KAFO with the hip and ankle linkage system decreased the horizontal rotation range of the pelvis and increased stride length [9]. These studies mostly focused on changes in joint angles and temporal parameters. Muscle contractions provide power for movements, and both kinematics and muscles interact with joint contact forces. Therefore, alternations in ankle joint motion may result in deviations of the associated muscle activity patterns and hip joint contact forces, which however no research studies examined.

The purpose of this study was to compare biomechanical parameters and muscle activities between people walking with combined knee and ankle immobilization and individual knee immobilization and evaluate the effects of ankle joint motion on these two gaits. The compared biomechanical parameters include kinematics of ankle, hip, and pelvis, net hip joint contact forces, and ground reaction forces. The muscle activities came from trunk and lower limb muscles. Due to the absence of ankle push-off mechanism and postural control in combined knee and ankle immobilization gait, we hypothesized that the ROM of pelvis and hip, thigh muscle activities, and hip contact forces in walking with combined knee and ankle immobilization would be greater than their counterparts in walking with individual knee immobilization. Given heterogeneous biomechanical performances of the patients with different impairments, this study started from the healthy controls walking in such two conditions, which is helpful to analyze the impact of ankle joint motion on kinematics and kinetics of other joints without interferences from great injury differences of patients.

## 2. Methods

### 2.1. Participants

Ten healthy individuals (6 men and 4 women; height 1.69 ± 0.11 m; weight 61.6 ± 11.8 kg; age 24.8 ± 2.3 years) were recruited in the study. Criteria for recruitment were (1) no cardiovascular disease, vestibular or respiratory disorder, uncorrected visual impairment, and motor neuron injury or neurological condition, (2) no lower extremity pain or lower extremity surgery within the past year, and (3) no history of ankle sprain. Approval was granted by the Institutional Review Board and the Ethics Committee. All participants signed an approved informed consent document before data collection.

### 2.2. Experimental Procedures

Trunk and lower extremity muscles on right side including the rectus abdominis (RA), obliquus externus abdominis (OE), erector spinae (ES), gluteus maximus (GMAX), gluteus medius (GMED), rectus femoris (RF), adductor longus (AD), semitendinosus (ST), tibialis anterior (TA), and gastrocnemius (GA) were measured. The electromyographic signals of these muscles were collected by a wireless surface electromyography (EMG) system (Telemyo DTS, Noraxon Inc., Scottsdale, AZ, USA). The signals were sampled at 1500 Hz, cut off at 1500 Hz, and amplified 1000 times. Pairs of Ag/AgCl surface electrodes (Tianrun Sunshine Medical Supplies Co., Ltd., China) were attached on the muscle bellies after cleaning the skin with alcohol wipes [10]. The maximum voluntary contraction (MVC) techniques included trunk flexion for RA, trunk upward bending for OE, trunk extension for ES, hip hyperextension for GMAX, hip abduction for GMED, knee flexion for ST, hip adduction for AD, knee extension for RF, ankle dorsiflexion for TA, and ankle plantar flexion for GA [11, 12]. The resistance was applied in opposite direction of the participant movement trends. The maximum value was recorded in three repeated MVC trials for one muscle. Different MVC movements were carried out in random order. Participants were allowed to rest for two minutes between two trials to avoid muscular fatigue.

**Figure 1:**
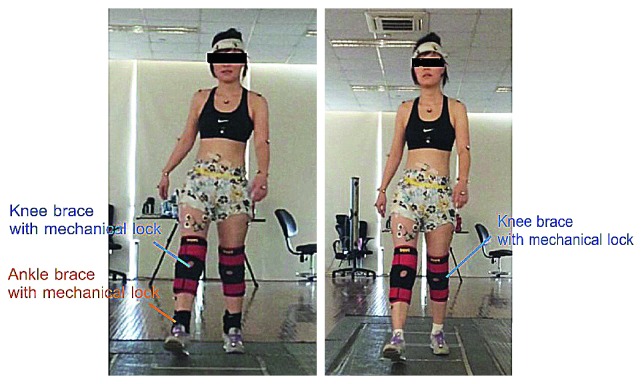
Two gaits were tested in this study. (a) Both knee and ankle immobilization (KAI) gait and (b) knee immobilization (KI) gait.

Participants were asked to walk along a 10 m walk away at their comfortable speeds in two gaits (i.e., individual knee immobilization (KI) and both knee and ankle immobilization (KAI)) (Figure 1). The braces used in this study incorporated mechanical locking feature to fix knee in full extension and ankle without plantar/dorsiflexion during walking. All participants were trained to walk in these two gaits for one week and 20 minutes per day for each gait before the study. After a week-long training, all participants reported that they were familiar with and have mastered these two gaits. The qualitative assessment from the participants was used to evaluate the sufficiency in attained skill level. Motion data and ground reaction force were collected by VICON motion capture system (Vicon Nexus v1.8.5, Oxford Metrics, Oxford, UK) at 100 Hz and two AMTI force platforms (Advanced Mechanical Technology Inc., USA) at 3000 Hz, respectively. The motion capture system, force platforms, and EMG system were synchronized to guarantee that all devices started recording data at the same time. Markers were placed according to Plug-in-Gait Marker Placement [13], and markers on knee and ankle joints were attached on the corresponding places on the braces. 6–8 strides on average for each trial and 5 valid trials for each condition were collected. The two walking conditions were carried out in random, and participants were allowed three minutes for rest between two conditions. All data were collected in the Gait and Motion Analysis Laboratory at the National Research Center for Rehabilitation Technical Aids (Beijing, China).

### 2.3. Musculoskeletal Model

**Figure 2:**
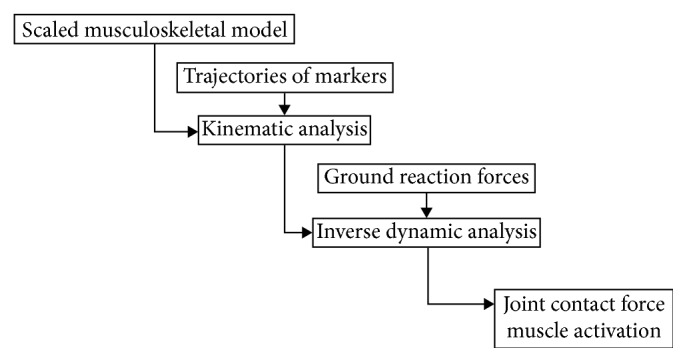
The procedure of joint contact force and muscle activation calculation in AnyBody software.

A generic, whole-body musculoskeletal model was built in AnyBody Software (AnyBody Modeling System v5.3, Model Repository v1.6, Aalborg, Denmark). This model had seven degrees of freedom for each lower limb (i.e., a revolute joint at the knee and two spherical joints at the hip and ankle, respectively) and six spherical lumbar joints between T12 and S1 with fixed centers of rotation [14]. The segments superior to the T12 joint were regarded as a single trunk segment. The generic model was scaled to each participant using anthropometric measurements. Experimental motion and ground reaction force data were input to drive the model and generate gait pattern. A least-squares optimization between virtual and experimental marker coordinates was used to best reproduce participants' measured motion [15]. Joint contact forces and kinematics were calculated in AnyBody Software and Vicon Nexus, respectively (Figure 2).

### 2.4. Data Analysis

Stride cycles were extracted from right heel strike to subsequent right heel strike. Contact forces between femur and acetabulum of the pelvis (hip joint contact force) were resolved along mediolateral, proximodistal, and anteroposterior axis and normalized to body mass. Envelopes of EMG data were extracted by using a 60 Hz high-pass filter, a full-wave rectifier, and a 10 Hz low-pass filter in sequence. All EMG data were processed with software (MR-XP 1.07 Master Edition) and normalized to corresponding MVC value. Times series of ankle, hip, and pelvis angle, hip joint contact forces, EMG envelope, and ground reaction forces were normalized to 0–100% of the gait cycle. Averages and ranges of each quantity were calculated in the first double support (DS1), single support (SST), second double support (DS2), and swing (SW) phase. A paired *t*-test was used to assess the differences (*α* = 0.05) between the two gait patterns in average and range of kinematic, joint contact force, ground reaction force, and EMG normalized activation during the four discrete phases. Correlations between experimental and simulated muscle activations from AnyBody system were carried out by the Pearson correlation coefficient. A correlation was considered significant when *p* < 0.05. The statistical calculations were performed with SPSS 22.0 software (SPSS Inc., Chicago, IL, USA), and *p* values (FDR method) for multiple comparisons were adjusted with *R* packages (*R* × 64 3.6.1). A difference was considered significant when corrected *p* < 0.05.

## 3. Results

### 3.1. Kinematics

**Figure 3:**
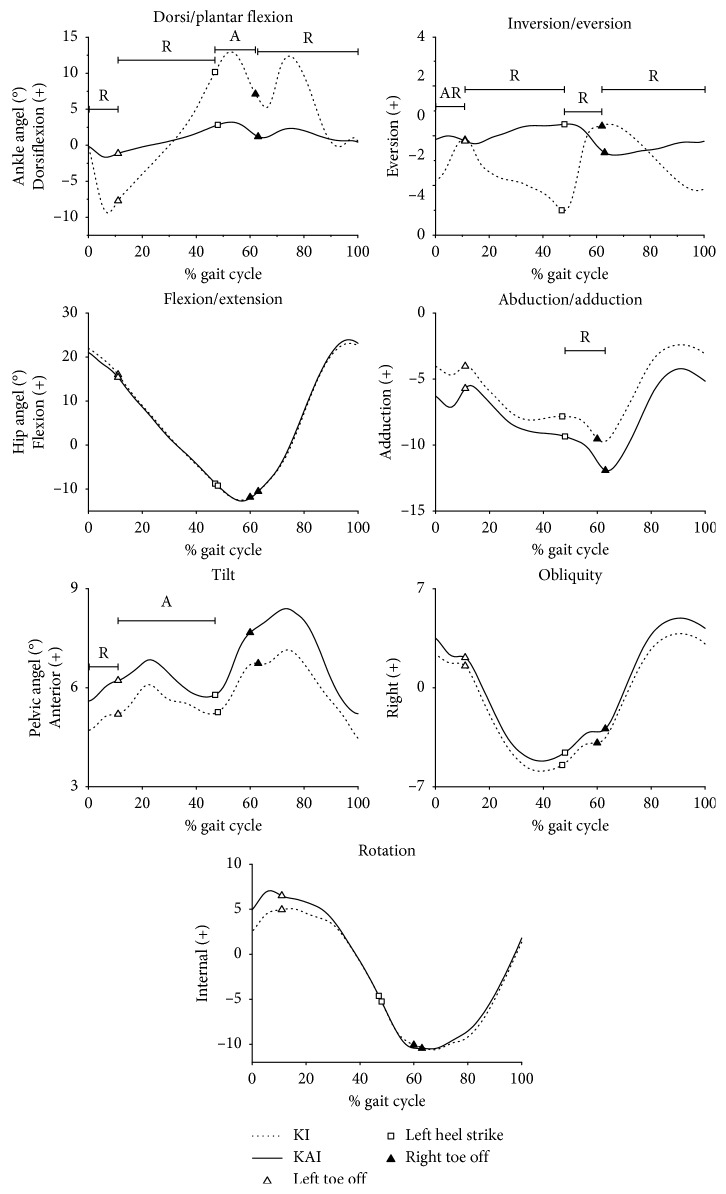
Group average results for average angle of ankle, hip, and pelvis of ten participants walking in two gaits. “A” means significant difference in average, and “R” means significant difference in range.

Figure 3 shows the average angle of ankle, hip, and pelvis for ten participants walking in KAI and KI gait in a gait cycle. Participants in KI gait had significantly greater ranges of ankle motion in the sagittal plane (*p* = 0.026, ＜0.001, ＜0.001 in DS1, SST, and SW phase, respectively) and the frontal plane (*p* = 0.03, ＜0.001, 0.025, and ＜0.001 in four discrete phases, respectively), and they had more dorsiflexion during the second double support (*p*=0.028) and more inversion during the first double support phase (*p*=0.042) (Table 1). Kinematic differences in hip were presented in a greater range and amplitude of pelvic anterior tilt during the first double support (*p*=0.006) and the single support phase (*p*=0.044), respectively. A significant increase was found in the hip abduction range during the second double support phase (*p*=0.015) in KAI gait (Table 1). The angular waveforms of hip flexion, pelvic obliquity, and rotation were generally similar between the two gaits.

### 3.2. Force

**Figure 4:**
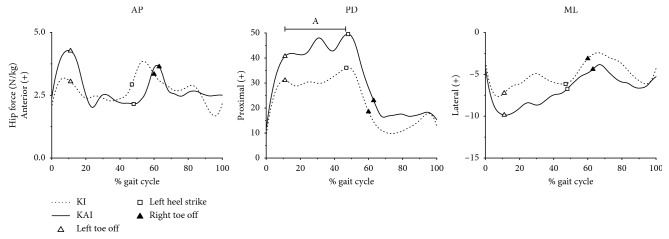
Group average results for the mediolateral, proximodistal, and anteroposterior component of average hip contact forces of ten participants walking in two gaits. AP is anteroposterior, PD is proximodistal, and ML is mediolateral.

Figure 4 presents the three components of average hip contact forces for ten participants walking in the two gaits. Most ranges and amplitudes of hip joint contact force components did not present significant differences between KAI and KI gait. The hip contact forces in proximodistal axis in KAI gait were significantly larger than their counterparts in KI gait during the single support phase (*p*=0.045). Peaks in anteroposterior and proximodistal component occurred nearly at the toe-off phase in the two gaits.

**Figure 5:**
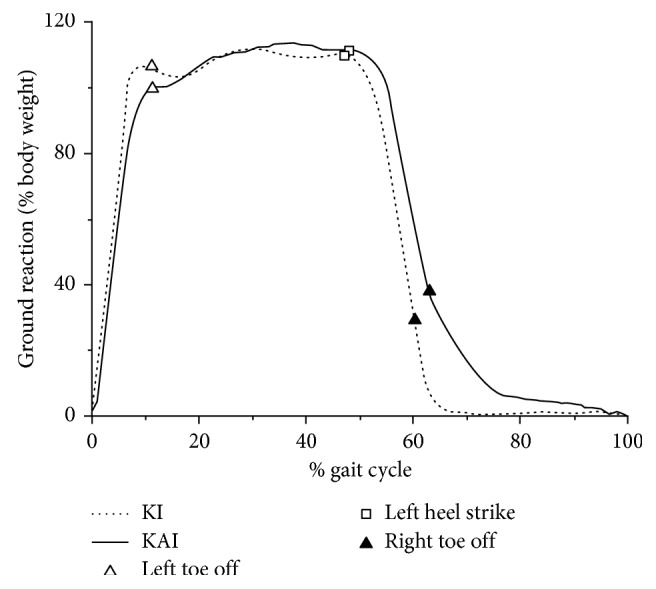
Group average results for the ground reaction forces of ten participants walking in two gaits.

Figure 5 shows the ground reaction forces for ten participants walking in the two gaits. The motion of ankle joint was not an influence factor on ground reaction force, since there were no significant differences between KAI and KI gait in range and amplitude throughout the entire gait.

### 3.3. Muscle Activities

**Figure 6:**
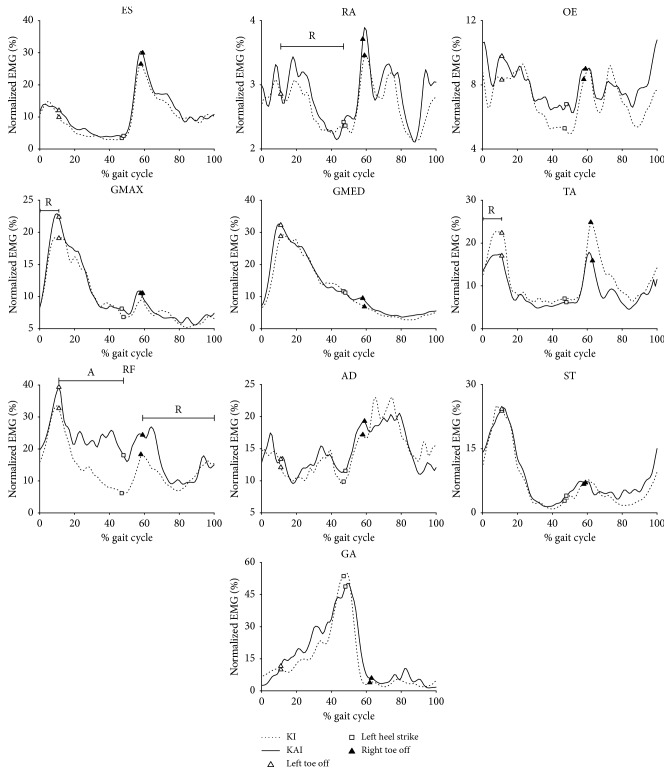
Group average normalized electromyography of trunk and lower limb muscles of ten participants walking in two gaits. RA, rectus abdominis; OE, obliquus externus abdominis; ES, erector spinae; GMAX, gluteus maximus; GMED, gluteus medius; RF, rectus femoris; AD, adductor longus; ST, semitendinosus; TA, tibialis anterior; GA, gastrocnemius.

Participants in KAI gait had numerous differences in normalized EMG of trunk and lower limb muscles across the gait cycle in comparison to KI gait (Figure 6 and Table 2). During the first double support phase, greater ranges of muscle activity were found within GMAX (*p*=0.044) in KAI gait and TA (*p*=0.044) in KI gait, respectively. During the single support phase, greater ranges of muscle activity within RA (*p*=0.015) and greater average normalized EMG amplitude within RF (*p*=0.045) were obtained. A greater range of muscle activity within RF (*p*=0.024) in KAI gait were presented during the swing phase.

**Figure 7:**
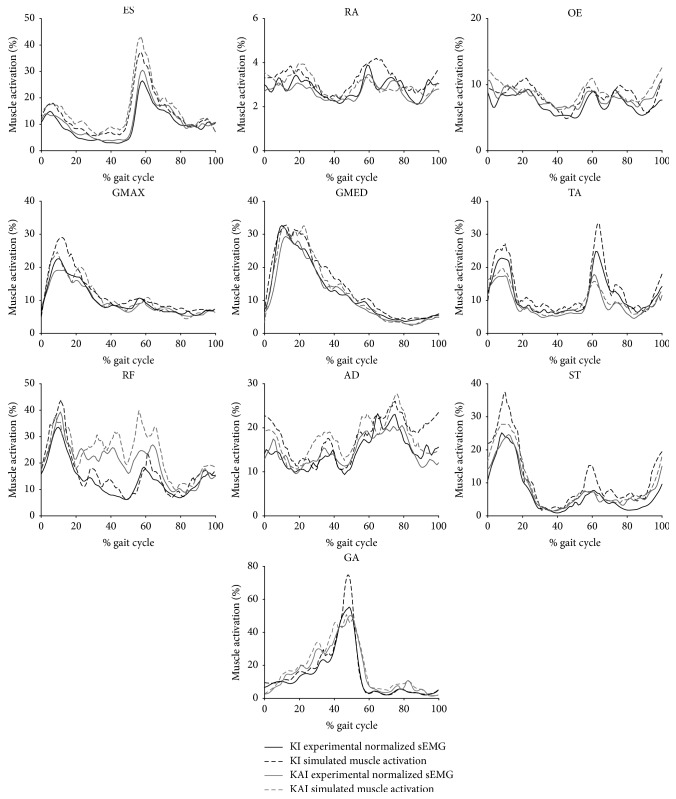
Simulated muscle activations and experimental EMG patterns of trunk and lower limb muscles of ten participants walking in two gaits.

Simulated muscle activations in two walking conditions were consistent with their experimental EMG patterns, as all tested trunk and lower limb muscle EMG patterns in KAI and KI gait were significantly correlated to corresponding simulated muscle activations (all *p* < 0.001) (Figure 7).

## 4. Discussion

The purpose of this study was to compare pelvis-hip biomechanics and trunk and lower limb muscle activities between people walking with combined knee and ankle immobilization and individual knee immobilization. Our results suggested that compared to the individual knee immobilization gait, the participants walking with combined knee and ankle immobilization increased pelvic tilt and hip abduction. These alternations in kinematics were associated with the hip contact force in proximodistal direction increasing and trunk and lower limb muscle activities changing. To our knowledge, this is the first study that investigated the effects of ankle joint motion on pelvis-hip biomechanics and muscle activity patterns in walking with knee immobilization.

A more rapid and an increased anterior pelvic tilt during the first double support and the single support phase, respectively, were found in the participants walking with KAI, which was likely to compensate for the absence of ankle motion in the sagittal plane [16]. There was a high coordination between ankle and hip muscle activities in human gait [17, 18]. In the first double support phase, GMAX provided vertical support and slowed forward progression in KI gait [18]. The coordination between GMAX and TA was impaired in KAI gait; therefore, greater muscle activity may be required in GMAX to compensate for lower force contributions by TA. The rectus femoris is a biarticular muscle which arises from the anterior inferior iliac spine and a groove superior to the acetabulum and ends into the base of the patella [19]. Donald Neumann summarized the kinesiology of the hip and indicated that “a sufficiently strong and isolated bilateral contraction of any hip flexor muscle will either rotate the femur towards the pelvis, the pelvis (and possibly the trunk) towards the femur, or both actions simultaneously” [20]. The proximal part of the rectus femoris plays an important role in pelvic tilt and hip flexion [21]. As the knee joints were locked in both KAI and KI gait, and the hip angles did not present significant differences in the single support phase, therefore, the increased RF and RA activation could provide the power for more anterior pelvic tilt in KAI gait.

During the second double support phase, the participants in KAI gait had a greater range of hip abduction in KAI in combination with less ankle inversion than in KI gait. This phase involved weight shifting in the mediolateral direction and interactions between the lower limb joints and musculatures to maintain balance in the frontal plane [22]. An increased range in hip abduction in KAI gait should have caused higher muscle activity in the GMED as the primary hip abductor. However, no significant difference was observed in statistical analysis, and the possible reason was that the gravity assisted hip abductors in accelerating the center of mass medially [23]. Compared to normal gait (mean maximum ankle inversion was about 0.5°) [24], the participants in KI gait had more ankle inversion throughout the entire gait, which was vulnerable to lateral ankle sprain.

A significant increase in the range of RF activity was found in KAI gait relative to KI gait in the swing phase. In KI gait, the soft tissue of ankle was stretched to generate passive ankle plantarflexor moment and enabled the absorption of energy during terminal stance and subsequent return of energy during the swing phase [25]. The main function of RF during walking is to extend the leg at the knee joint and flex the hip joint [26]. The participants adopted to increase power generated by hip flexor (i.e., RF) as a compensation for the absence of passive-elastic mechanism at the ankle joint in KAI gait. Additionally, this difference may also relate to the increased lower limb inertia moment about the hip axis due to the ankle brace in KAI gait in which greater RF activity contributed to accelerating the swing leg.

Kinematics affected the joint contact forces, and previous studies reported that increases in hip abduction and pelvic hike were related to hip contact forces increasing [27], which could explain that the average values of proximodistal component in AKI gait were much greater than those in KI gait during the single support phase. The maximum proximodistal and anteroposterior force of normal gait were 3.94 and 1.06 times body weight, respectively [28]. However, the peak values of proximodistal component in AKI gait and anteroposterior component in both AKI and KI gait were out of normal range. This phenomenon may increase the risk of hip joint injuries and pain.

The results of the study provided insights into the differences of pelvis-hip biomechanics and muscle activity patterns in KAI and KI gait and have some implications for the orthosis/brace design intended to constrain ankle plantar/dorsiflexion. The ankle immobilization gait did not alter the muscles that span the ankle joint too much (except TA). Instead, RA and RF that seem to have no direct relationship to ankle joint were affected significantly. The increase in muscle activation and ROM during walking is likely to cause muscle fatigue, joint wear or pain, and even gait change after healing. These phenomena may prompt the engineers to consider passive degree of freedom or provide suitable stiffness for ankle joint of the orthoses and braces to prevent the potential adverse effects.

There were some methodological limitations in this study that should be taken into account. First, the sample size was low and 10 healthy participants may limit the external validity to the clinical environment as these participants did not suffer motor neuron injuries, quadriceps weakness, or postsurgical procedures. Individuals with impairments should be recruited in the future for further studies involving the effects of ankle joint motion on knee immobilization gait and the comparisons between healthy controls and individuals with impairments in biomechanical performance. Second, only EMG data of superficial trunk and lower limb muscles were collected. Correa et al.'s [29] studies suggested that deep muscles such as the iliopsoas and piriformis also play important roles in hip contact forces and body motion, which were not considered in this study. Third, the hip contact forces were not decomposed into individual muscle group contributions. Thus, it was difficult to determine the effects of an individual muscle on hip contact forces in KAI and KI gait, precisely.

## 5. Conclusion

This study investigated the pelvis-hip biomechanics and trunk and lower limb muscle activity patterns between healthy individuals walking with combined knee and ankle immobilization and individual knee immobilization and evaluated the effects of ankle joint motion on these two gaits. Compared to KI gait, healthy individuals walking in KAI gait increased the range and average angle of anterior pelvic tilt during the first double support and the single support phase, respectively, and the range of hip abduction during the second double support phase. These kinematic alternations caused changes in trunk and lower limb muscle activity patterns and a significant increase in proximodistal component of hip contact forces during related gait phases. This study revealed significant differences in pelvis-hip biomechanics and trunk and lower limb muscle activity patterns between the two gaits. The findings may prompt further understanding of the effects of ankle joint on braced gait and provide some references to develop the novel orthoses and braces.

## Figures and Tables

**Table 1:** Mean (SD) of joint angles that were significantly different (^*∗*^) or that approached significance in average or range during a phase of gait.

Joint motion	Phase	Quantity	KAI	KI	*p* value
Ankle dorsi/plantar flexion	DS1	RNG	1.81 (0.93)	10.21 (8.65)	0.026^*∗*^
	SST	RNG	3.89 (1.86)	18.40 (7.71)	<0.001^*∗*^
	DS2	AVG	2.38 (2.00)	9.88 (8.43)	0.028^*∗*^
	SW	RNG	2.05 (0.78)	13.30 (2.06)	<0.001^*∗*^
Ankle inversion/eversion	DS1	AVG	−0.09 (2.15)	−0.96 (4.59)	0.042^*∗*^
		RNG	0.21 (1.49)	1.64 (3.65)	0.030^*∗*^
	SST	RNG	0.68 (2.73)	2.25 (4.13)	<0.001^*∗*^
	DS2	RNG	1.15 (1.57)	2.00 (6.93)	0.025^*∗*^
	SW	RNG	0.56 (2.06)	2.68 (4.06)	<0.001^*∗*^
Pelvis tilt	DS1	RNG	1.80 (0.74)	0.83 (0.38)	0.006^*∗*^
	SST	AVG	7.51 (3.16)	6.15 (3.75)	0.044^*∗*^
Hip adduction/abduction	DS2	RNG	2.75 (2.38)	1.96 (2.60)	0.015^*∗*^

DS1, the first double support; SST, single support; DS2, the second double support; SW, swing phase; AVG, average; RNG, range.

**Table 2:** Mean (SD) of muscle activities that were significantly different (^*∗*^) or that approached significance in average or range during a phase of gait.

Muscle	Phase	Quantity	KAI	KI	*p* value
RA	SST	RNG	3.37 (0.83)	1.51 (0.47)	0.015^*∗*^
GMAX	DS1	RNG	12.55 (4.29)	10.44 (3.32)	0.044^*∗*^
RF	SST	AVG	23.69 (6.21)	15.48 (10.64)	0.045^*∗*^
	SW	RNG	13.91 (3.43)	11.31 (2.56)	0.024^*∗*^
TA	DS1	RNG	6.01 (6.75)	10.43 (7.26)	0.044^*∗*^

## Data Availability

The data used to support the findings of this study are included within the article.
